# Supplementary material for: High resolution cryo EM analysis of HPV16 identifies minor structural protein L2 and describes capsid flexibility
Source: Sci Rep. 2021 Feb 10;11:3498. doi: 10.1038/s41598-021-83076-5 (PMC7876116; doi:10.1038/s41598-021-83076-5)
Supplement: Supplementary file 1 — Supplementary Information. [file 41598_2021_83076_MOESM1_ESM.pdf]

High resolution cryo EM analysis of HPV16 identifies minor structural protein L2 and describes capsid flexibility

Daniel J. Goetschius[1,2*], Samantha R. Hartmann[1*], Suriyasri Subramanian[2], Carol Bator[1], Neil D. Christensen[3,4,5], Susan L Hafenstein[1,2**]

[1]Department of Biochemistry and Molecular Biology, Penn State University, University Park, PA 16802, USA

[2]Department of Medicine, Penn State University College of Medicine, Hershey, PA 17033, USA

[3]Department of Pathology, Pennsylvania State University College of Medicine, Hershey, PA 17033, USA

[4]The Jake Gittlen Laboratories for Cancer Research, Hershey, PA 17033, USA

[5] Department of Microbiology and Immunology, Pennsylvania State University College of Medicine, Hershey, PA 17033, USA

* Equal contribution

** Corresponding: shafenstein@psu.edu

Supplementary Information

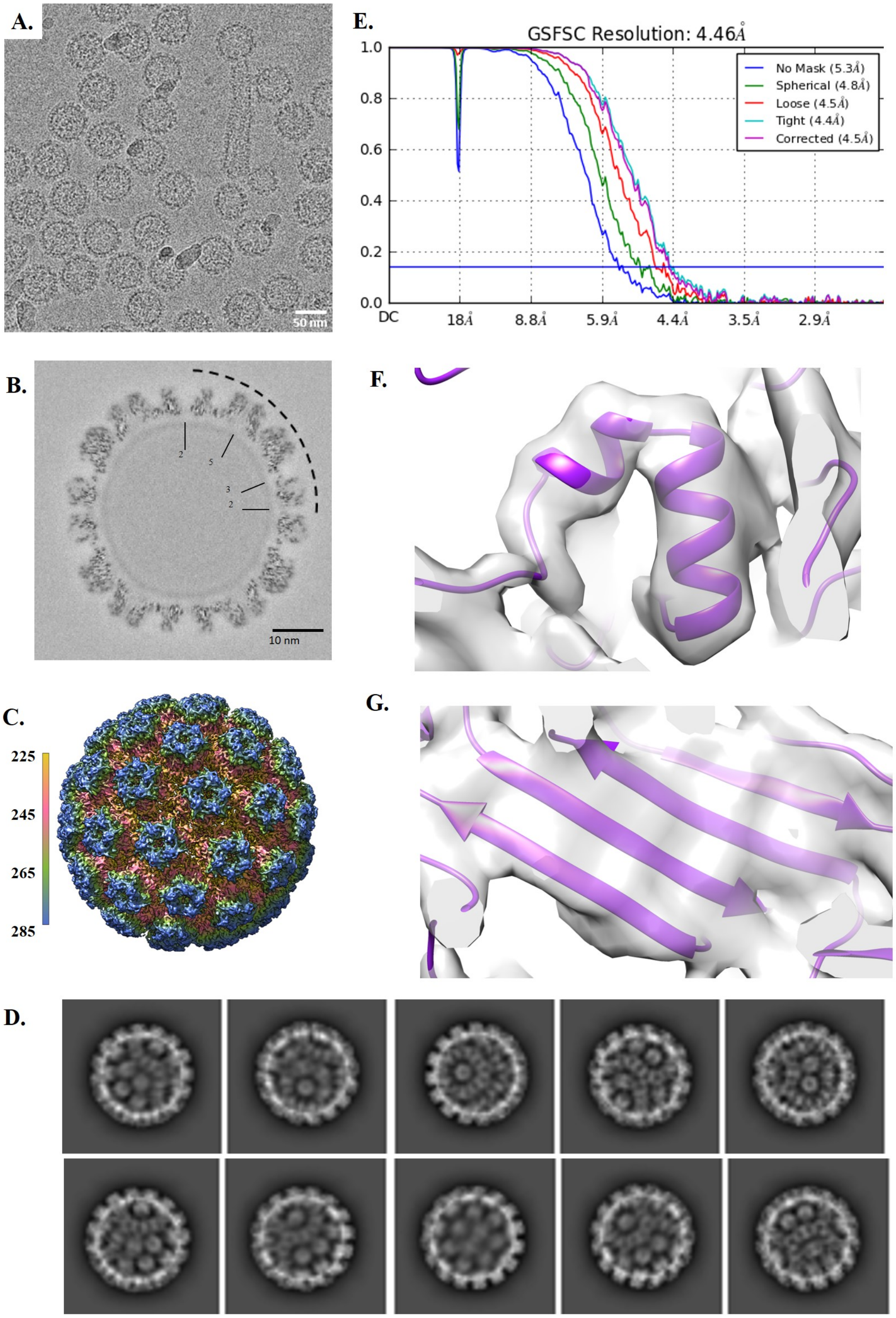

SFig 1. Icosahedral Reconstruction. (A) Representative micrograph 1,164 out of 8,936 collected. (B) Central cross-section of icosahedral structure with the symmetry labeled in the dotted region. (C) Full icosahedral reconstruction colored radially in Angstroms. (D) 2D classes that were used for icosahedral reconstruction. (E) FSC Curve for icosahedral refinement. (F) Representative alpha helix (amino acid residues: 385-394, 396-401) at 4.5Å. (G) Representative beta sheet (amino acid residues: 71-76, 335-325, 153-160, 254-248) at 4.5Å.

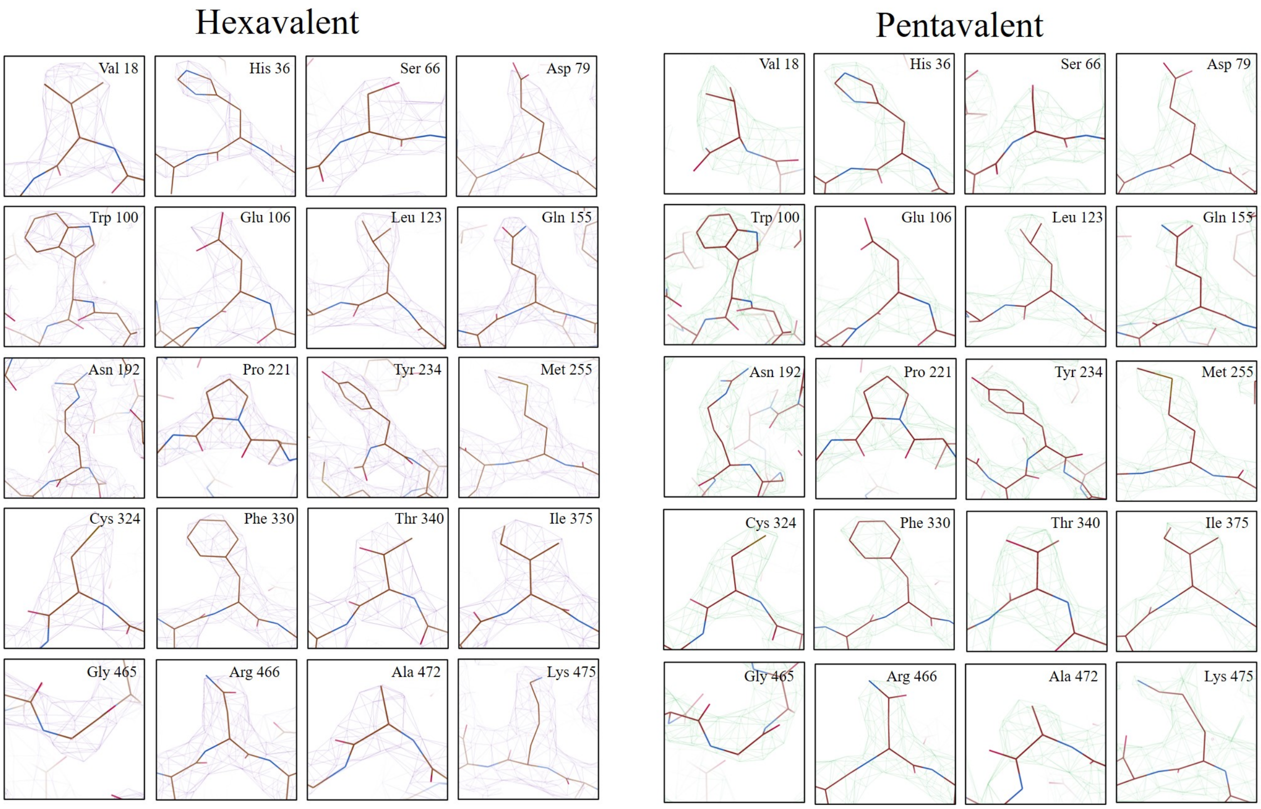

SFig 2. Representative density of all side chains in pentavalent and hexavalent capsomers. Representative side chains were chosen from L1 (brown) and compared between the pentavalent (purple density) and hexavalent (green density) capsomer environments.

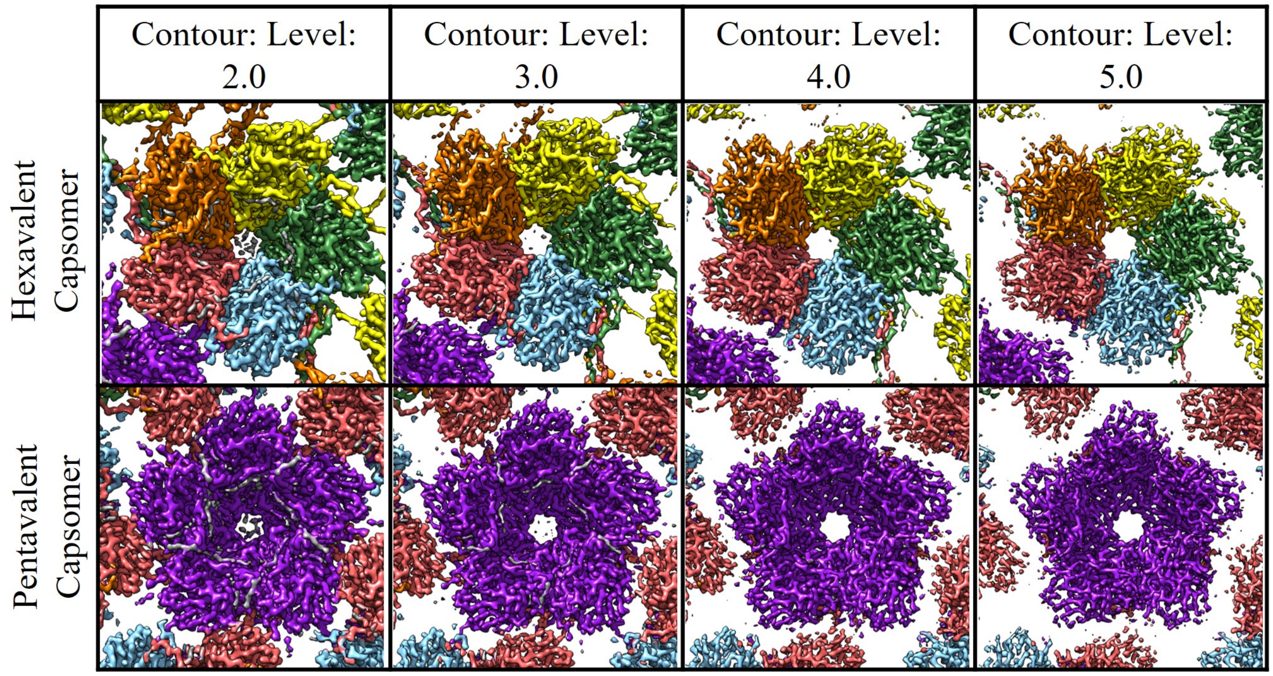

SFig 3. L2 Density. The surface rendered hexavalent (top) and pentavalent (bottom) capsomer density maps were colored as in Fig. 1A within 2Å of the L1 protein chain with unfilled density (gray) corresponding to L2. The L2 density is stronger in the pentavalent capsomer as can be seen with the changing contour of the map. In the hexavalent capsomers the same internal density can be noted, but the density disappears along with noise. Two distinct areas of gray density can be seen that are correlated to each chain of L1 in the capsomer.

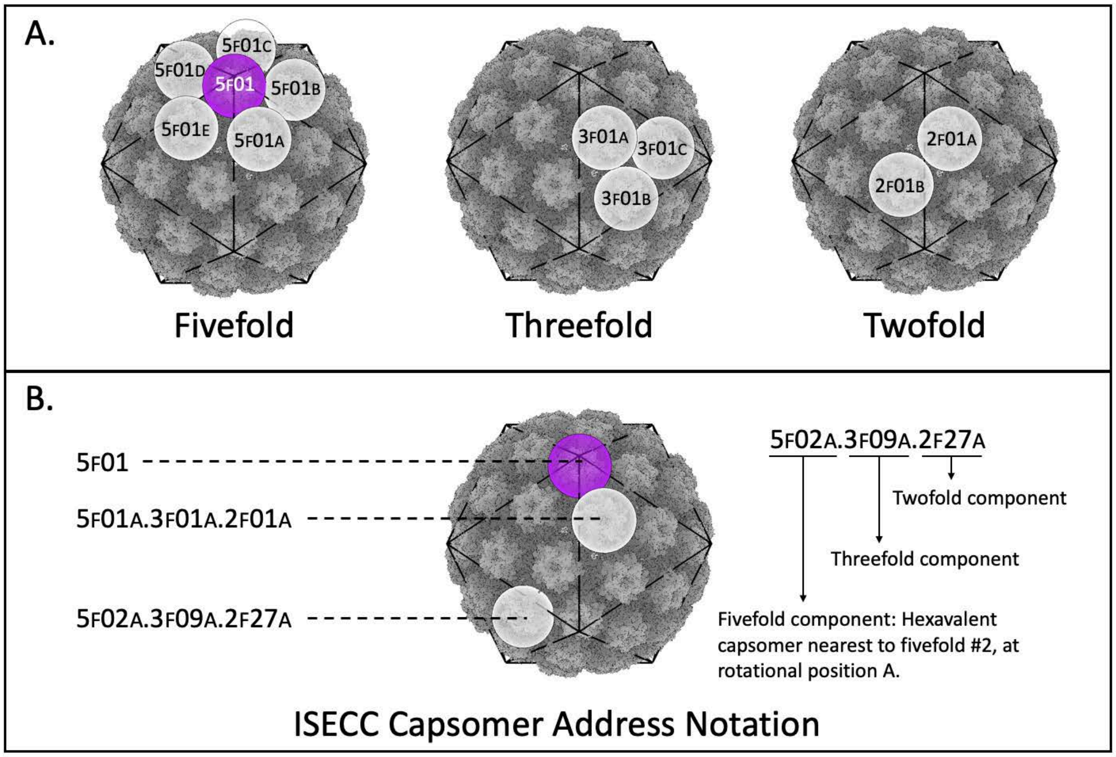

SFig4. Capsomer addresses assigned in ISECC. A) Each capsomer is assigned an address identifying the nearest symmetry vertices. Pentavalent capsomers only have a fivefold designation without any rotational parameter (left, purple). Hexavalent capsomers receive designations for the nearest fivefold (left), threefold (center), and twofold (right) axis, as well as rotational parameters. B) Examples of complete addresses implemented in ISECC are shown as one-part (pentavalent) and three-part (hexavalent) capsomer designations. Addresses are assigned during subparticle generation after normalization of the input vectors to a standard, shared asymmetric unit. This allows refinement parameters for any given subparticle to be correlated with other subparticles from the same parental particle.

Supp. Table 1. Cryo-EM data collection, refinement and validation statistics

Data Collection and Processing	Icosahedral	Pentavalent Capsomer	Hexavalent Capsomer
Magnification	59,000	59,000	59,000
Voltage (kV)	300	300	300
Electron Exposure (e-/Å^2)	60	60	60
Defocus Range (um)	0.5-3.0	0.5-3.0	0.5-3.0
Pixel Size (Å)	1.1	1.1	1.1
Symmetry Imposed	I1	C5	C1
Micrographs Collected	10,143	-	-
Micrographs Rejected (Bad Ice)	1,207	-	-
Micrographs Accepted	8,936	-	-
Initial Particle Number	202,705	-	-
Final Particle Number	181,299	181,299	181,299
Subparticles per Particle	-	12	60
Final Subparticle Number	-	2,175,588	10,877,940
Map Resolution (Å)	4.46	3.15	3.08
FSC Threshold	0.143	0.143	0.143

Refinement	Recombined Icosahedral Asymmetric Unit
Model composition	
Non-hydrogen atoms	22492
Protein Residues	2864
B-Factors	
Protein	-
R.m.s. Deviations	
Bond Length (Å)	0.006
Bond Angles (°)	1.025
Validation	
MolProbity Score	2.65
Clash Score	13.17
Rotamer Outliers (%)	5.10
Ramachandran Plot	
Favored (%)	92.18
Outliers (%)	0.46

Supplemental Table 2. Sequence Alignment of Ser306 – Ile328 Loop Region

HPV Type	Overall Sequence Percent Identity	Sequence Percent Identity of Loop	Sequence
16	-	-	SDAQIFNKPYWLQRAQGHNNGI
31	82.97%	92.3%	SDAQIFNKPYWMQRAQGHNNGI
52	76.82%	100%	SESQLFNKPYWLQRAQGHNNGI
58	76.23%	100%	SESQLFNKPYWLQRAQGHNNGI
33	79.60%	100%	SESQLFNKPYWLQRAQGHNNGI
11	68.83%	92.3%	SEAQLFNKPYWLQKAQGHNNGI
6	68.59%	92.3%	SEAQLFNKPYWLQKAQGHNNGI
45	65.51%	84.6%	SDSQLFNKPYWLHKAQGHNNGI
18	65.87%	84.6%	SDSQLFNKPYWLHKAQGHNNGV

Supplemental Table 3: Custom ISECC metadata

**Metadata label**	**Example**	**Description**
rlnImageOriginalName	000004@ {micrographname}.mrcs	Existing metadata label. Repurposed to carry the identifier for the particle image from which a subparticle was derived.
rlnCustomUID	subparticleUID_ 000000001	Sequential, unique value identifying each subparticle
rlnCustomVertexGroup	Pentavalent:<br>5f08<br><br>Hexavalent:<br>5f08c.3f02b.2f27a	Pentavalent capsomers are given a numerical value for the 5f symmetry axis on which they lay. There are 12 unique options for this metadata label.<br><br>Hexavalent capsomers are designated by the nearest 5f, 3f, and 2f symmetry axis, as well as as a letter (a-e, a-c, a-b) designation the counter-clockwise rotation order about the given symmetry axis. There are 60 unique options for this metadata label.
rlnCustomRelativePose	0.809,<br>+0.309i,<br>-0.500j,<br>+0.000k	Capsomer orientation relative to icosahedrally-refined capsid, in quaternion format
rlnCustomOriginXYZ AngstWrtParticleCenter	-125.7926, 15.1589, -102.4911	Capsomer origin relative to icosahedrally-refined capsid, in Å
